# Improving public health laboratory preparedness at European Union level: learning points from experiences during the COVID-19 pandemic

**DOI:** 10.2807/1560-7917.ES.2026.31.32.2600017

**Published:** 2026-08-13

**Authors:** Sarah Parkinson, Saoirse Moriarty, Fifi Olumogba, Katarina Pisani, Daniela Moye Holz, Daniela Rodriguez Rincon, Cyril Barbezange, Eeva K Broberg

**Affiliations:** 1 RAND Europe, Cambridge, United Kingdom; 2 European Centre for Disease Prevention and Control (ECDC), Stockholm Sweden

**Keywords:** public health, infectious disease, preparedness, laboratories, pandemic, infectious disease

## Abstract

This Perspective discusses how European Union (EU)-level action could support public health laboratories in preparing for future threats from respiratory infectious diseases. It draws extensively from a study that looked at the experience of public health laboratories in EU/ European Economic Area (EEA) countries during the COVID-19 pandemic. This study included a literature review and consultation with stakeholders from public health laboratories in EU/EEA countries and from EU institutions. We discuss how to address the challenges faced by public health laboratories during the COVID-19 pandemic, focusing particularly on the role of EU institutions. The Perspective concludes by discussing the importance of improving public health laboratory preparedness for future health threats when there is no ongoing public health emergency, instead of during a health emergency.

## Background

The COVID-19 pandemic and recent avian influenza and influenza A outbreaks have reinforced the importance of preparing for future respiratory health threats. Considering this, the European Centre for Disease Prevention and Control (ECDC) and other European Union (EU) institutions have identified pandemic preparedness as a priority in improving health, resilience and security in the EU [1-3]. Public health laboratories (PHLs) play an important role in pandemic preparedness and response, providing expert advice, developing national standards and protocols and identifying and characterising pathogens.

This Perspective discusses how EU-level action could support PHLs in preparing for future threats from respiratory infectious diseases and argues that more should be done to prepare for the next health emergency when there is no ongoing public health emergency. While it reflects the views of the authors, it draws extensively from a study looking at the experience of PHLs in EU and European Economic Area (EEA) countries during the COVID-19 pandemic. This study was published as an ECDC technical report [4], and included a literature review, along with consultations with individual stakeholders from PHLs in EU/EEA countries and with stakeholders from EU institutions (ECDC, the Health Emergency Preparedness and Response Authority (HERA), the Directorate-General for Health and Food Safety (DG SANTE), the European Food Safety Authority (EFSA), the European Medicines Agency (EMA), and the World Health Organisation (WHO) Regional Office for Europe). Many of the recommendations proposed here are based on what participants from PHLs in EU/EEA countries reported that they expected from EU-level institutions. However, the scope of the published ECDC technical report [4] was limited to PHLs; other actors involved in public health response during the COVID-19 pandemic were not consulted. Therefore, this Perspective does neither capture the viewpoint of all relevant stakeholders in public health, such as specialists in medical diagnostics, hospital laboratory staff or epidemiologists, nor does it consider the response of public health institutions that are distinct from PHLs in EU/EEA countries. In addition, the ECDC technical report [4] focused on challenges and how PHLs addressed these challenges during the pandemic. As such, it is not complete in its description of the EU’s role during health emergencies.

We here first discuss the EU’s role in addressing uncertainty during public health emergencies. Next, we examine EU-level efforts to support EU/EEA countries in managing capacity challenges during the COVID-19 pandemic, as well as efforts to support collaboration and data sharing. Finally, we discuss the role of EU institutions in maintaining advances that were made during the pandemic and learning from the pandemic to improve preparedness.

## Uncertainty during public health emergencies

The initial phase of the COVID-19 pandemic, and the year 2020 in particular, were characterised by uncertainty, particularly regarding the transmission dynamics and the epidemiology of the severe acute respiratory syndrome coronavirus 2 (SARS-CoV-2) virus [5]. Public health laboratories faced challenges because of the limited availability of detection tools, the variable performance of assays and a lack of standardised methods across laboratories [6]. They also faced challenges in obtaining positive samples and control material during the initial phases of the pandemic in January and February 2020, when only a few cases and isolated clusters had been detected in Europe. This limited access made it difficult to develop and validate assays at a time when rapid action was crucial. Although real-time RT-PCR (RT-qPCR) detection protocols were published in January 2020 and used in PHLs [7], their use in clinical laboratories remained limited until commercial kits entered the market. These factors decreased the capacity of countries to detect the virus and track its spread. Improvements were only seen once commercial kits became widely accessible and once the growing range of methods and techniques underwent external quality assessment (EQA). External quality assessments help ensure that laboratories use reliable tests for detecting circulating pathogens, and, in a health emergency, enable the use of multiple assays which can reduce dependency on a single supplier. A robust EQA programme with clear assay acceptance criteria and appropriate control materials is therefore essential [8,9].

Public health laboratories faced further uncertainty around the different roles they were expected to fulfil during the pandemic, which made it challenging to know what balance of activities was needed to most effectively respond to the pandemic. The pandemic pushed many PHLs to adopt new and expanded roles, including spending more time supporting and providing EQA services for hospital, diagnostic and other types of laboratories and conducting additional activities such as assessing population immunity through seroepidemiological testing. Representatives from PHLs in EU/EEA countries also reported spending more time communicating with policymakers and the public during the COVID-19 pandemic than in previous health emergencies. They reported that they were unsure how to carry out this role effectively while also completing their other responsibilities and explained that communication was particularly challenging because of the politicisation and misinformation early in the pandemic.

In addition, representatives from PHLs reported uncertainty about what types of data from PHL activities are most needed to inform public health decision-making at different points during pandemics and in the aftermath. For example, they reported being unsure about the amount of genomic sequencing required for decision-making at different stages. During the COVID-19 pandemic, PHLs faced a high demand for testing, with genomic sequencing introduced into workflows. They explained that while genomic surveillance was necessary to detect and monitor the spread of variants and to develop diagnostic tools and vaccines, this could have been achieved with a lower volume of sequencing. Some participants attributed the high demand for sequencing to political pressure, potentially due to media coverage of variants. Questions regarding how much genomic sequencing is required and how it should be implemented in practice (e.g. metadata, linkages, international data sharing, turnaround time) have been raised since early in the pandemic [10,11]. While some evidence is available, optimal strategies depend on a wide range of factors which have not yet been consolidated.

The consultation with public health laboratories highlighted roles that the EU-level institutions could play in reducing the uncertainty faced by laboratories during the pandemic (Box 1).

Box 1 **Role of the European Union level in mitigating uncertainty in future health emergencies, according to public health laboratories in European Union and European Economic Area countries [4]**

**Collecting evidence to inform best practices**

European Union (EU) institutions have a role in collecting evidence to inform best practices during uncertain periods in public health emergencies. The European Commission is a major funder of research through initiatives such as Horizon Europe. Participants highlighted areas they saw as a priority for research funding, including the development of diagnostics and assays, research on best practices in laboratory methods and techniques and on ‘right sizing’ surveillance activities.

**Providing guidance, including on external quality assessment and standards, for laboratory tests, along with implementation support**

European Union-level guidance and training can help align methods between different laboratories and improve the ability to combine data and data types from different EU/EEA countries by improving standardisation and metadata quality. It can help reduce uncertainty, increase uptake of evidence-based practices and enhance skills and capabilities. The support can include laboratory training, EQAs, virus characterisation and diagnostic assay development, as provided by the ECDC-funded AURORAE (lAboratory sUppoRt fOr influenza and SARS coRonAvirus 2 for Europe) consortium during the COVID-19 pandemic [28]. However, efforts to improve standardisation must balance the potential risks of relying on a limited number of methods that are standardised across laboratories: viral mutations could reduce test accuracy [29]; standardised methods may miss patients with atypical immune responses [30]; key laboratory supplies may become unavailable (e.g. due to supply chain disruptions), reducing testing capacity during emergencies.

**Providing specific support regarding additional functions during public health emergencies, beyond public health laboratories’ usual responsibilities**

European Union-level institutions can publish guidance on how PHLs should balance providing core PHL services and engaging with other types of laboratories. Similarly, EU-level support can also help upskill PHL leadership in communication during public health emergencies; EU institutions can produce training materials and guidance documents, gather evidence on what works and continue to facilitate knowledge-sharing opportunities for PHLs to exchange learning points from the COVID-19 pandemic.

ECDC: European Centre for Disease Prevention and Control; EEA: European Economic Area; EQA: external quality assessment; PHL: public health laboratory.

## Capacity constraints

The COVID-19 pandemic led to increased demand for PHL services. Public health laboratories faced significant capacity constraints due to workforce challenges, limited physical and digital infrastructure and a lack of supplies, often struggling to keep up with surges in demand during the pandemic. In particular, there was a high demand for genomic sequencing, which required specific skills and infrastructure that some laboratories did not have [12], particularly in the early months of the pandemic. The requirements to work under biosafety level 3 (BSL3) reduced the number of institutes able to perform isolation and virus neutralisation assays. While these capacity challenges were more commonly faced by smaller EU/EEA countries, they were seen in all countries.

Digital infrastructure emerged as a critical component of pandemic preparedness, enabling data storage, sharing, linkage and integration of data across multiple sources. However, participants noted the limited capacity of existing systems to handle large amounts of data as well as challenges with data sharing. Many countries implemented new digital infrastructures to cope with data from the pandemic [13-15], but the need to build new solutions while stabilising existing systems was challenging for public health systems [16].

Skills, infrastructure and capacity will likely continue to be a challenge in future public health emergencies. However, lessons from the COVID-19 pandemic can help in understanding what PHL activities are essential for a proper response and how different surveillance mechanisms (e.g. wastewater and environmental surveillance) can be implemented most effectively to support PHLs during a pandemic. In addition, there are important lessons from the COVID-19 pandemic on what types of data management systems should be kept in place and further developed during peacetime to allow for data to be rapidly and securely shared with all relevant stakeholders during emergencies.

The participants outlined roles that the EU institutions could play in supporting laboratories as they strengthen their capacities to respond to health threats (Box 2).

Box 2 **Role of the European Union level in increasing capacity, according to public health laboratories in European Union and European Economic Area countries [4]**

**Supporting capacity and preparedness in European Union and European Economic Area countries through funding with programmes such as EU4Health and HERA Invest**

Directing funding and support to help fill capacity gaps in PHLs, including workforce and physical and digital infrastructure gaps, can help improve preparedness for future threats. Such funding is especially important for smaller and less well-resourced countries.

**Facilitating joint procurement and stockpiling of key laboratory supplies**

European Union-level institutions can improve access to medical countermeasures and other laboratory supplies, including diagnostic kits, reagents, consumables, reference materials for priority pathogens and personal protective equipment. This can occur through joint procurement frameworks among EU/EEA countries as well as programmes such as EU4Health and rescEU [31] in combination with efforts to improve domestic manufacturing capabilities of certain supplies within the EU.

**Producing evidence and guidelines to help public health laboratories prioritise competing demands for capacity**

Public health institutes and PHLs need to understand, for example, how much resources they should spend on genomic sequencing and asymptomatic testing for a specific pathogen in future pandemics, which will fluctuate depending on the pathogen characteristics and the pandemic stage. Similarly, PHLs will need to know when and how participatory, wastewater, environmental surveillance or pooled sampling can be used most efficiently with other forms of surveillance.

**Providing services and information at a European Union level**

As one example, since the publication of the ECDC Technical Report [4], several EU Reference Laboratories for public health (EURLs) have been designated by the European Commission. The EURLs will develop and disseminate reference diagnostics and standardised test protocols, provide reference materials, conduct pathogen characterisation, EQAs and provide scientific advice, training and technical assistance [32].

ECDC: European Centre for Disease Prevention and Control; EEA: European Economic Area; EQA: external quality assessment; EU: European Union; PHL: public health laboratory.

## Collaboration and data sharing

During the COVID-19 pandemic, PHLs drew on national networks, networks across EU/EEA countries [17,18] and broader international collaborations [6,19,20] to share data, samples, capacity and best practice. Participants reported that these networks were important in supporting PHL pandemic response and helping coordinate between PHLs, other types of laboratories and wider stakeholders in public health. Public health laboratories drew on existing and new networks during the COVID-19 pandemic, which were especially important in coordinating sequencing and supporting genomic data sharing. Examples of networks that helped support collaboration and knowledge exchange during the COVID-19 pandemic include the European COVID-19 surveillance network (ECOVID-Net), European COVID-19 reference laboratory network (ECOVID-LabNet) and the Network of National Public Health Institutes (NNPHI) [21,22].

Public health laboratories also collaborated with different sectors to fill capacity gaps during the COVID-19 pandemic [18]. Public health laboratories regularly procured services, laboratory equipment and consumables from private companies. Moreover, during the COVID-19 pandemic, public health stakeholders and private companies collaborated more extensively, allowing PHLs to draw on equipment, expertise and capacity held in the private sector, including from companies outside traditional providers. Public health laboratories also drew on capacities from other sectors, including food safety, the veterinary sector, the military and academia, to cope with demand. While working across sectors helped address challenges that laboratories faced during the pandemic, knowledge about contact points, regulation, ways of working and data and capacity across different sectors was required.

Encouraging collaboration between One Health areas (human, animal and plant health) is important, particularly due to the zoonotic origin of many viruses and the role that agriculture and the environment play in the spread of infectious diseases. Continued efforts to harmonise and integrate surveillance data from different sectors are needed.

The COVID-19 pandemic highlighted challenges in accessing and sharing data and biological samples crucial for effective disease surveillance and response [23]. These challenges include practical barriers, regulatory and privacy concerns, and infrastructural gaps. To better prepare for public health emergencies and to speed up access to data and samples, clear guidance on how data and materials can be shared, digital infrastructure and shared data standards are critical, both nationally and internationally. For example, the WHO’s Investigations and Studies Global Initiative (Unity Studies) protocols for COVID-19 helped to tailor laboratory seroepidemiological investigation and antibody assays to local contexts [24].

The discussion identified roles that EU-level institutions could play in enhancing coordination during crisis situations (Box 3).

Box 3 **Role of the European Union level in improving collaboration, according to public health laboratories in European Union and European Economic Area countries [4]**

**Strengthening existing networks to support preparedness for public health emergencies across European Union and European Economic Area countries and internationally**

Continued support to ECDC laboratory networks and other European and international collaboration mechanisms can help improve preparedness for future public health emergencies. One example is the United4Surveillance network that was established in 2022 which includes 40 partners across Europe and focuses on integrating existing and new data sources for more comprehensive EU/EEA infectious disease surveillance, prevention and control [33].

**Coordinating capacity across sectors during public health emergencies**

To do this effectively, there is a need to consolidate information on the capacities, knowledge, supplies and data that are held across different sectors. It is important to set up these mechanisms for collaboration outside public health emergencies and EU-level support can help ensure that relationships do not need to be built from scratch during future public health emergencies.

**Helping European Union and European Economic Area countries understand how regulation might affect pandemic preparedness (e.g. data sharing and medical countermeasure development)**

For example, there is a need for more EU-level support in understanding how General Data Protection Regulation (GDPR) applies to sharing samples, data and metadata during a pandemic, and in clarifying the impact of the In Vitro Diagnostic Regulation (IVDR) (Regulation (EU) 2017/746) on in-house test development, and procurement and rapid implementation of in vitro diagnostic medical devices during a public health emergency. This concern was recently highlighted in the literature [34].

ECDC: European Centre for Disease Prevention and Control; EEA: European Economic Area; EU: European Union.

## Maintaining advances and learning from the COVID-19 pandemic

While PHLs faced many challenges during the COVID-19 pandemic, there were also major advances made in the tools and methods available for surveillance. For example, genomic surveillance advanced rapidly and capacity for sequencing and analysis grew [19,25]. In addition, new approaches to surveillance were implemented across EU/EEA countries including wastewater and other environmental surveillance, the widespread use of rapid antigen detection tests and home and community sampling [15,26]. These methods have the potential to improve preparedness and response in the future if they are used effectively, particularly for detection and in the early phase of a pandemic. In addition, further developments, such as metagenomic surveillance, may also continue to improve the ability to rapidly identify emerging novel pathogens. There are also valuable lessons from the COVID-19 pandemic that can improve preparedness in PHLs in the future. For example, the COVID-19 pandemic confirmed the importance of timely information in responding to public health threats and the role that adaptability and flexibility play in pandemic preparedness and response. For instance, it is important that PHLs are prepared to implement rapidly shifting testing strategies and laboratory procedures based on emerging evidence. It is also important that procurement processes and regulation are adaptable enough to support flexible responses to challenges faced during public health emergencies.

The COVID-19 pandemic also confirmed the importance of planning and rehearsing preparedness so that capacity can be scaled up rapidly during public health emergencies in a way that allows for rapid and effective response to threats. This is a common reflection after public health emergencies occur and participants reflected on the danger of political will and funding diminishing in the aftermath of the COVID-19 pandemic.

The participants described roles that EU-level institutions could play in consolidating and disseminating lessons from the COVID-19 pandemic to guide future emergency responses (Box 4).

Box 4 **Role of the European Union level in learning from the COVID-19 pandemic, according to public health laboratories in European Union and European Economic Area countries [4]**

**Improving preparedness for cross-border threats through planning and preparedness exercises**

European Union (EU) institutions have a role in supporting EU/EEA countries to create and implement evidence-based preparedness plans describing how stakeholders should scale up capacity and coordinate during future emergencies. Preparedness and simulation exercises help PHLs and others involved in detecting and responding to threats to identify weaknesses, pathways for coordination and ways to improve detection and response mechanisms.

**Clarifying the role that different EU institutions play in preparedness**

Study participants from EU institutions highlighted potential overlaps in their roles. Clarifying these roles would allow for smoother collaboration and coordination during public health emergencies and help improve trust in the support being provided at EU level.

EEA: European Economic Area; PHL: public health laboratory.

## Conclusion

The COVID-19 pandemic highlighted the critical role of PHLs in responding to pandemics and other health emergencies. This Perspective, informed by findings from a study published in an ECDC technical report [4], highlights the challenges faced by PHLs during the COVID-19 pandemic and identifies solutions and opportunities for maintaining advances and improving preparedness for future health threats.

Pandemics are a serious cross-border threat, for which EU institutions have clear and urgent responsibilities. European Union-level actors are uniquely placed to improve collaboration across EU/EEA countries, to help address gaps in capacity in low-resource settings that increase the risk for other countries, to improve the evidence base to respond to future pandemics and to develop and help implement best practice guidance. Findings of the study indicated that EU-level support is critical for preparedness and that EU-level action is expected.

In the aftermath of public health emergencies, it is likely that political will and funding decline when sustained funding is needed to maintain the technological advances and retain trained staff [27]. With limited resources, it is even more important to be able to detect threats efficiently. To form best practice around efficient surveillance, evidence is required on which types of surveillance are needed at different points during health emergencies.

Threats from infectious diseases, novel pathogens, zoonotic diseases and antimicrobial resistance are on the rise, due in part to climate change, habitat destruction and the inappropriate use of antimicrobials. These threats sit within a wider context of risks caused by chemical, biological, radiological and nuclear (CBRN) threats, which are a high priority on the EU agenda. Improving health security at EU level requires action to improve preparedness and coordination during health emergencies. Taking these steps to better prepare now, rather than waiting until the next pandemic occurs, will help improve responses.

## Data Availability

Data sharing is not applicable to this perspective as no datasets were generated or analysed in writing this article.
